# Community Composition, Antifungal Activity and Chemical Analyses of Ant-Derived Actinobacteria

**DOI:** 10.3389/fmicb.2020.00201

**Published:** 2020-02-11

**Authors:** Zhiyan Wang, Zhiyin Yu, Junwei Zhao, Xiaoxin Zhuang, Peng Cao, Xiaowei Guo, Chongxi Liu, Wensheng Xiang

**Affiliations:** ^1^Key Laboratory of Agricultural Microbiology of Heilongjiang Province, Northeast Agricultural University, Harbin, China; ^2^State Key Laboratory of Phytochemistry and Plant Resources in West China, Kunming Institute of Botany, Chinese Academy of Sciences, Kunming, China; ^3^State Key Laboratory for Biology of Plant Diseases and Insect Pests, Institute of Plant Protection, Chinese Academy of Agricultural Sciences, Beijing, China

**Keywords:** ant-derived actinobacteria, actinobacterial community, high throughput sequencing, antifungal activity, phytopathogenic fungi, agroactive compound

## Abstract

Actinobacteria associated with insects represent one potentially rich source of novel natural products with antifungal activity. Here, we investigated the phylogenetic diversity and community composition of actinobacteria associated with ants using a combination of culture-dependent and -independent methods. Further, we assessed the antagonistic activity against phytopathogenic fungi and identified the secondary metabolites from isolates with bioactivity. A total of 416 actinobacterial isolates were obtained from three ant species (*Camponotus japonicus*, *Lasius fuliginosus*, and *Lasius flavus*) located in five nests. The largest amount of isolates were observed in the head samples. 16S rRNA gene sequencing showed that the isolates were diverse and belonged to ten genera within the phylum *Actinobacteria*, with *Streptomyces* and *Micromonospora* comprising the most abundant genera. High-throughput sequencing analyses revealed that the actinobacterial communities were more diverse and dominated by the families *Nocardioidaceae*, *Nocardiaceae*, *Dermacoccaceae*, *Intrasporangiaceae*, and *Streptomycetaceae*. In addition, 52.3% of the representative isolates had inhibitory properties against phytopathogenic fungi. Chemical analysis of one *Streptomyces* strain led to the discovery of two known compounds and one new compound. These results demonstrated that ant-derived actinobacteria represented an underexplored bioresource library of diverse and novel taxa that may be of potential interest in the discovery of new agroactive compounds.

## Introduction

The phylum *Actinobacteria* consists of a wide range of Gram-positive bacteria with high guanine+cytosine (G+C) content. Members of the phylum are the most important sources of antimicrobials, and have generally been isolated from terrestrial and marine habitats (Qin et al., 2016; Lee et al., 2018). Recently, insect-associated actinobacteria have been intensely studied for their capacity to produce structurally novel natural products with antimicrobial properties. For example, dentigerumycin A, a new depsipeptide that contains unusual amino acids (β-hydroxyleucine, *N*-hydroxyalanine, piperazic acid and γ-hydroxypiperazic acid) and a pyran side chain, isolated from an symbiotic *Pseudonocardia* sp. associated with fungus-growing ant, *Acromyrmex octospinosus*, shows selective antifungal activity (Oh et al., 2009a). Selvamicin, a new polyene macrolide with antifungal activity, was produced by two *Pseudonocardia* isolates recovered from nests of fungus-growing ant (*Acromyrmex dentigerum*) (Van Arnam et al., 2016). Mycangimycin, a structurally novel polyene peroxide with pronounced antifungal and antimalarial activity, was secreted by a symbiotic *Streptomyces* sp. associated with the southern pine beetle (*Dendroctonus frontalis*) (Oh et al., 2009b). Further studies of the antinobacterial symbionts of the southern pine beetle led to the discovery of antifungals frontalamides A and B, two novel polycyclic tetramate macrolactams (Blodgett et al., 2010). Macrotermycins A and C, two novel glycosylated polyketide macrolactams with selective antifungal activity, were produced by *Amycolatopsis* sp. M39 isolated from the fungus-growing termite species *Macrotermes natalensis* (Beemelmanns et al., 2017). Sceliphrolactam, a novel polyene macrocyclic lactam with antifungal activity against amphotericin B-resistant *Candida albicans*, was isolated from a wasp-associated *Streptomyces* sp. (Oh et al., 2011). Further, a recent study assessed insect-associated *Streptomyces* strains as a source of new antimicrobials by application of genomics, metabolomics, and bioactivity assays. Their results showed that *Streptomyces* from insects harbored many of uncharacterized biosynthetic gene clusters which led to the discovery of new molecules, and exhibit notably greater activity against fungi than soil-derived *Streptomyces* (Chevrette et al., 2019). These findings highlighted that chemical studies of insect-associated actinomycetes would be an effective strategy for the discovery of new chemotypes with antimicrobial activity.

Insect-associated actinobacteria have been isolated from diverse insects, including fungus-growing ants, fungus-growing termites, southern pine beetle, dung beetle, beewolf digger wasps, honeybee and grasshoppers (Currie et al., 1999; Scott et al., 2008; Patil et al., 2010; Kroiss et al., 2010; Visser et al., 2012; Kim et al., 2013). *Streptomyces* and *Pseudonocardia* are the most predominant genera cultivated. Other genera, including *Amycolatopsis*, *Nocardiopsis*, *Nocardia*, *Saccharothrix*, *Kitassatospora*, and *Propionicimonas*, were also identified (Patil et al., 2010; Zucchi et al., 2011; Matarrita-Carranza et al., 2017; Van Arnam et al., 2018). With over five million species, insects spread to nearly all terrestrial niches on our planet, and host numerous chemically prolific bacteria (Stork et al., 2015; Van Arnam et al., 2018). However, there is still limited information related to the chemical ecology of insect-associated actinobacteria. Thus, insects can be regarded as a huge and underexplored reservoir for unusual microorganisms with antimicrobial potential.

Fungi are the oldest enemies of terrestrial organism. For example, the earliest terrestrial plants probably developed chemicals to counteract their assault (Swain, 1978). Ants are widespread in most terrestrial habitats. However, the damp underground nests in which they live were also suitable for fungal growth (Bailey, 1920), which leads to a high risk of fungal infestation for their larvae and food. To counteract these threats, they have developed a range of defensive strategies. For example, fungus-growing ants cultivate actinobacteria that produce antibiotic substances, which can protect the fungal gardens against the antagonistic fungus (Oh et al., 2009a; Carr et al., 2012). Further, Sánchez-Peña et al. (2008) found that antinobacteria associated with fungus-growing ants showed significant inhibitory activity against plant pathogenic fungi, which broadens the studies of antinobacteria insect associates to control economically important plant pathogens.

Ants are an very diverse group of insects with largely untapped antinobacterial communities (Matarrita-Carranza et al., 2017). Here, we investigated the diversity of actinobacteria from three ant species (*Camponotus japonicas*, *Lasius fuliginosus*, and *Lasius flavus*) using culture-dependent and culture-independent methods, assessed the anti-phytopathogenic fungal activity of cultured isolates, and identified the secondary metabolites produced by one *Streptomyces* strain with broad-spectrum antifungal activity.

## Materials and Methods

### Sample Collection

**FIGURE 1:**
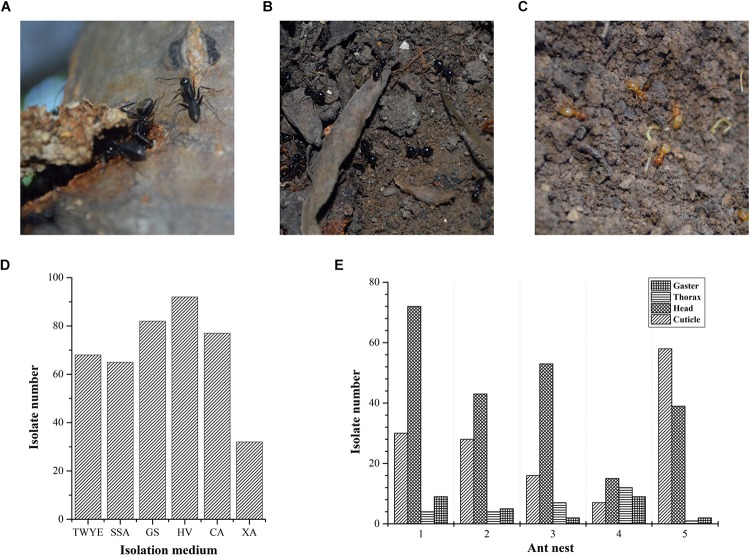
Ant species and actinobacteria isolation. **(A)** *Camponotus japonicas*; **(B)** *Lasius fuliginosus*; **(C)** *Lasius flavus*; **(D)** Compositions of the six different media used for the isolation of actinobacteria from ant samples; **(E)** Actinobacteria isolated from different body parts of ants from five nests.

Ant colonies were collected from the campus of Northeast Agriculture University located in Harbin, Heilongjiang, north China (45°44′ N, 126°43′ E) in September 2015. Three ant species, including *C. japonicas* (Formicinae, Figure 1A), *L. fuliginosus* (Formicinae, Figure 1B) and *L. flavus* (Formicinae, Figure 1C), were sampled from five nests. *C. japonicas* colonies were caught from nests 1, 2, and 4. Nests 1 and 2 were constructed under pine trees, whereas nest 4 was on the oak tree. *L. fuliginosus* and *L. flavus* colonies were obtained from nests 3 and 5, which were built under willow tree and oak tree, respectively. Samples from each nest were collected using sterile forceps and deposited into a pre-sterilized container. Then, they were brought to the lab and processed immediately.

### Actinobacteria Isolation

For large sized ant (*C. japonicas*), five individuals were placed in an autoclaved 1.5 mL Eppendorf tube for actinobacteria isolation. For medium (*L. fuliginosus*) and small sized ants (*L. flavus*), ten and twenty individuals were used, respectively. Each sample was rinsed twice with sterile water to remove the surface soil and adherent epiphytes, followed by immersion in 1.2 mL sterile water and vortexed for 30 s. The suspension was transferred to a new autoclaved Eppendorf tube for actinobacteria isolation from the cuticle. Subsequently, the ant samples were surface sterilized by 70% ethanol for 1 min. Ethanol waste was removed and ant specimens were rinsed three times with sterile water. After external sterilization, the specimens were divided into head, thorax and gaster, and each body part was separately immersed in 1.2 mL sterile water and cultivated for 30 min at 28°C with shaking at 180 rpm. Finally, an aliquot of 200 μL suspension was plated onto six different actinobacteria-selective media types: humic acid-vitamin (HV) agar (Hayakawa and Nonomura, 1987); Gause’s synthetic (GS) agar no. 1 (Atlas, 1993); chitin agar (CA) (Hanshew et al., 2015); tap water-yeast extract (TWYE) agar (Qin et al., 2009); sodium succinate-asparagine (SSA) agar (Li et al., 2012); and xylan-arginine (XA) agar (Qin et al., 2012). All media were amended with nalidixic acid (20 mg/L) and cycloheximide (50 mg/L) to suppress the growth of gram-negative bacteria and fungi. Cultures were incubated at 28°C for 2–3 weeks. Colonies obtained after incubation were transferred onto oatmeal agar (ISP3) (Shirling and Gottlieb, 1966), and then maintained on slants at 4°C or as glycerol suspensions (20%, v/v) at −80°C.

### Taxonomic Identification of Isolates

Isolates were cultivated on ISP 3 medium at 28°C for 2 weeks, and then preliminarily identified according to their phenotypic characteristics including the characteristics of colonies on plates, color of aerial and substrate mycelium, and production of diffusible pigment. Genomic DNA was extracted from isolates using lysozyme-SDS-phenol/chloroform method (Maniatis et al., 1982). PCR amplifications for the 16S rRNA gene sequence were performed using the universal bacterial primers, 27F and 1492R (Lane, 1991). The reaction conditions were 95°C for 5 min, followed by 30 cycles of 95°C for 30 s, 55°C for 45 s, and 72°C for 1 min, with a final extension at 72°C for 10 min. The PCR products were purified and cloned into the vector pMD19-T (Takara) for sequencing. The almost full-length 16S rRNA gene sequences (∼1500 bp) were obtained and neighbor-joining phylogenetic tree was constructed using the molecular evolutionary genetics analysis (MEGA) software version 7.0 (Kumar et al., 2016). Bootstrap replication (1000 replications) was used to assess the topology of the phylogenetic tree (Felsenstein, 1985). The obtained gene sequences were deposited in the GenBank database under accession numbers KP784763-KP784808, KX777578-KX777633, KX977397-KX977399, and KR261651-KR261652, respectively.

### Culture-Independent Community Analysis

Five ant individuals (*C. japonicas*) were rinsed three times with sterile water, and then ground-up using a sterile pestle inside the tube. Total community DNA was extracted using FastDNA^®^SPIN for soil kit refering to the manufacturers’ specifications (MP Biomedicals, Solon, CA, United States). The V6 hypervariable region of the 16S rRNA gene was targeted for amplification by PCR with primers 967F and 1046R (Huse et al., 2008). The reaction conditions were 94°C for 2 min, followed by 30 cycles of 94°C for 30 s, 57°C for 30 s, and 72°C for 30 s, with a final extension at 72 for 5 min. PCR amplicon was purified using GeneJET Gel Extraction Kit (Thermo Scientific, Fermentas, Germany). Sequencing library was generated using NEB Next Ultra DNA Library Prep Kit for Illumina (New England Biolabs, Ipswich, MA, United States) according to the manufacturer’s instructions and index codes were added. The library quality was evaluated on the Qubit@ 2.0 Fluorometer and Agilent Bioanalyzer 2100 system (Agilent Technologies, Palo Alto, CA, United States). Finally, the library was sequenced with an Illumina HiSeq 2500 using 100 bp paired-end reads.

Raw reads were demultiplexed and quality-filtered using QIIME version 1.17 (Caporaso et al., 2010). Clean reads obtained were spliced using the PEAR software (Zhang et al., 2014). The merged sequences were then removed chimeras and clusterd in operational taxonomic units (OTUs) with the software UCLUST (Edgar, 2010) based on 97% nucleotide similarity. Taxonomy of the representative sequence for each OTU was assessed by RDP_Classifier and comparison against the SILVA 16S rRNA gene database (Quast et al., 2013). Raw sequencing data can be retrieved from the NCBI Short Read Archive under accession number SRR8061509.

### *In vitro* Antifungal Assays

Antifungal screening was performed against 13 different phytopathogenic fungi: *Rhizoctonia solani*, *Fusarium oxysporum*, *Curvularia lunata*, *Corynespora cassiicola*, *Setosphaeriaturcica turcicaf*, *Colletotrichum orbiculare*, *Alternaria solani*, *Helminthosporium maydis*, *Sphacelotheca reiliana*, *Sclerotinia sclerotiorum*, *Phytophthora sojae*, *Phytophthora capsici*, and *Phytophthora infestans*. All pathogens were maintained at the Key Laboratory of Agricultural Microbiology within the Heilongjiang Province, China. *P. sojae*, *P. capsici*, and *P. infestans* were incubated on carrot agar (Cao et al., 2017) at 20°C, whereas the others were incubated on potato dextrose agar (PDA) (Cao et al., 2017) at 28°C except *S. sclerotiorum* at 20°C. Antifungal activity of isolates were assessed using the dual culture plate assay (Hamzah et al., 2018). When a clear inhibitory zone had formed, typically within 1–2 weeks after fungal inoculation, the inhibition diameters were measured, and then the inhibition rates were calculated according to the formula described by Liu et al. (2019). The assay was performed three replicates.

### Secondary Metabolite Characterization

Five isolates with broad-spectrum antifungal activity, including three novel species *Streptomyces capitiformicae* 1H-SSA4, *Streptomyces amphotericinicus* 1H-SSA8 and *Streptomyces lasiicapitis* 3H-HV17(2), as well as strains *Streptomyces* sp. 1H-GS5 and *Streptomyces* sp. 1H-XA2, were performed for secondary metabolites characterization. The isolation and identification of secondary metabolites from strains 1H-SSA4, 1H-SSA8, 3H-HV17(2) and 1H-GS5 have been reported by our previous studies (Liu et al., 2016; Cao et al., 2017; Liu et al., 2017; Ye et al., 2017; Jiang et al., 2018). Here, we describe the detailed isolation and structural determination of the compounds from strain 1H-XA2.

Strain 1H-XA2 was grown on ISP 3 slant medium for 1 week at 28°C. Then it was inoculated into 250 mL baffled Erlenmeyer flasks containing 50 mL of sterile seed medium (TSB) and cultivated for 2 days at 30°C with shaking at 250 rpm. After that, aliquots (12.5 mL) of the culture were transferred into 1 L baffled Erlenmeyer flasks filled with 250 mL of the production medium (soluble starch 2%, tryptone 2%, glycerol 1%, NaCl 0.05%, K_2_HPO_4_⋅3H_2_O 0.05%, MgSO_4_⋅7H_2_O 0.05%, FeSO_4_⋅7H_2_O 0.05%, KNO_3_ 0.1%, pH 7.2–7.4) and cultured at 30°C for 1 week with shaking at 250 rpm. The fermentation broth (25 L) was centrifuged (4000 rev/min, 20 min), and the supernatant was extracted with ethylacetate three times. The ethylacetate extract was subsequently evaporated *in vacuo* to afford 10.0 g of oily crude extract. The mycelia were extracted with methanol (1 L), and then concentrated *in vacuo* to remove the methanol to yield the aqueous concentrate. This aqueous concentrate was finally extracted with EtOAc (1 L × 3) to give 1.0 g of oily crude extract after removing the ethylacetate. Both extracts revealed an identical set of metabolites based on HPLC and TLC analyses, and therefore, they were combined for further purification.

The crude extract (11 g) was subjected to silica gel using a successive elution of petroleum ether/ethylacetate (1:0, 10:1, 5:1, 1:1 and 0:1, v/v) (1-7). Fraction 6 was subjected to Sephadex LH-20 column elution with methanol to collect three fractions (F6A, F6B, and F6C). F6B was purified by semipreparative HPLC (YMC-Triart C_18_ column, 250 × 10 mm i.d., 5 μm, 3.0 mL min^–1^, 0–26.0 min CH_3_OH: H_2_O = 44:56, 26.1–39.0 min CH_3_OH: H_2_O = 62:38, 39.1–43 min CH_3_OH: H_2_O = 100:0) to obtain compound **1** (*t*_R_ = 38.2, 5.4 mg). Fraction 7 was subjected to Sephadex LH-20 column elution with MeOH to collect two fractions (F7A and F7B). Compound **2** (*t*_R_ = 11.5 min, 33.7 mg) and **3** (*t*_R_ = 18.5 min, 24.8 mg) were obtained from the F7A by semipreparative HPLC (YMC-Triart C_18_ column, 250 mm × 10 mm i.d., 5 μm, CH_3_OH: H_2_O = 48:52, 0.1% acetic acid, 3 mL/min).

Structures of compounds **1**, **2**, and **3** were determined using spectroscopic analysis. NMR spectra were measured with a Bruker Avance III-400 spectrometer in DMSO using TMS as internal standard. HR-ESI-MS or ESI-MS data were obtained using an UPLC-IT-TOF mass instrument (Shimadzu, Japan).

## Results

### Isolation of Ant-Derived Actinobacteria

A total of 416 isolates were obtained from three different ant species sampled and the most frequently isolated genus based on phenotypic characteristics was *Streptomyces* (∼80% of isolates). Of the five ant nests sampled, nest 1 yielded the most isolates (115), followed by nest 5 (100), nest 2 (80), nest 3 (78), and nest 4 (43). HV medium yielded the highest number of isolates (Figure 1D). The majority of isolates were cultured from head samples (222 isolates, 53.4%), followed by cuticle (139 isolates, 33.4%), thorax (28 isolates, 6.7%) and gaster (27 isolates, 6.5%) (Figure 1E). Based on morphological and cultural characteristics, 107 strains with different phenotypic characteristics were chosen for further investigation.

### Diversity of Cultured Actinobacteria

**FIGURE 2:**
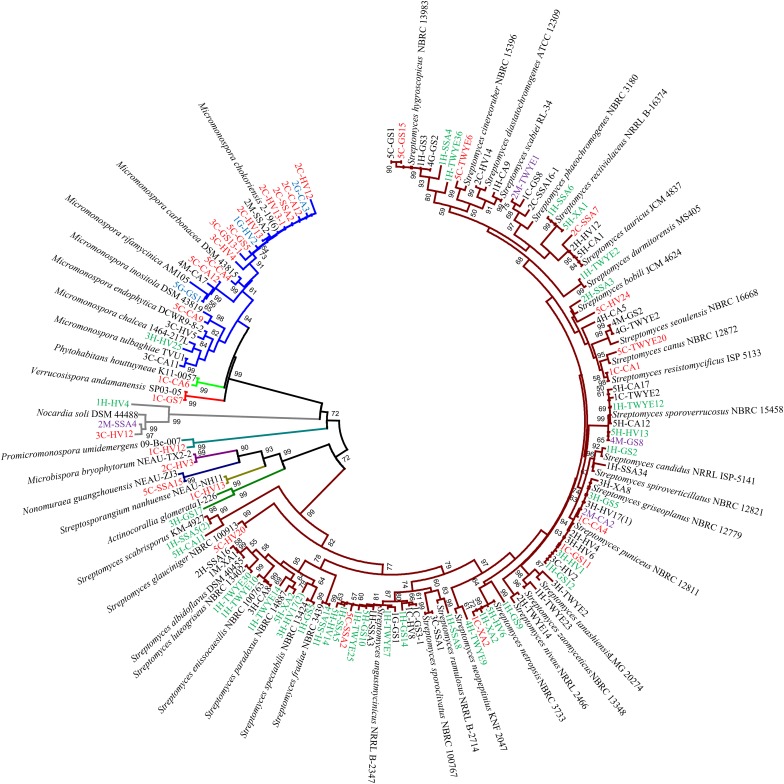
Neighbor-joining phylogenetic tree of 16S rRNA gene sequences from 107 actinobacteria in this study and their phylogenetic neighbors. Numbers at nodes are bootstrap values (percentages of 1000 replications); only values >50% are shown. A branch indicated by the same color belongs to the same genus. Isolates indicated by green from head, red from curticle, purple from thorax, and blue from gaster.

16S rRNA gene sequence analysis of the 107 isolates revealed considerable diversity, which distributed among six family: *Streptomycetaceae*, *Micromonosporaceae*, *Promicromonosporaceae*, *Nocardiaceae*, *Sreptosporangiaceae*, and *Thermomonosporaceae* within the phylum *Actinobacteria*, including ten known genera. *Streptomyces* was the most frequently isolated genus (73%, 77 isolates), followed by *Micromonospora* (19 isolates) and *Nocardia* (3 isolates). Some rare genera, including *Promicromonospora*, *Non-omuraea*, *Verrucosispora*, *Phytohabitans*, *Streptosporangium*, *Microbispora*, and *Actinocorallia* were isolated less frequently and were represented by only one strain (Figure 2 and Supplementary Table S1). Furthermore, EzTaxon analysis of the 16S rRNA gene sequences revealed that many strains showed relatively low similarities to the type strains of the corresponding genera. Finally, 13 strains, including 1C-GS8, 2C-SSA16(2), 1C-HV12, 1H-GS9, 1H-HV4, 1H-SSA4, 1H-SSA8, 2C-HV3, 2H-TWYE14, 3C-HV12, 3H-GS17, 3H-HV17(2), and 5H-CA11, were chosen for detailed identification using standard polyphasic taxonomic identification methods, and have been proposed to represent novel species of the genera *Streptomyces*, *Nocardia*, *Promicromonospora*, *Microbispora*, and *Actinocorallia* (Bai et al., 2016; Guo et al., 2016; Han et al., 2016; Liu et al., 2016a,b,c, 2018; Li et al., 2016; Cao et al., 2017; Piao et al., 2017; Ye et al., 2017; Jiang et al., 2018).

### Culture-Independent Community

**FIGURE 3:**
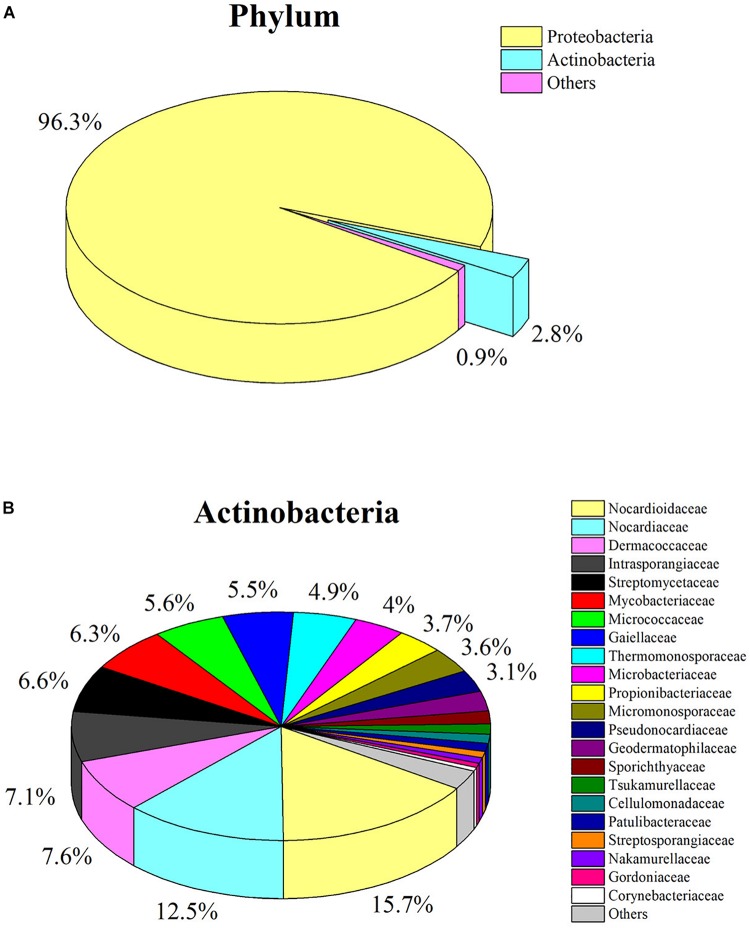
Analysis of culture-independent microbial communities. **(A)** Pie chart showing relative abundance of OTUs (>0.5%) at phylum level; **(B)** Pie chart showing relative abundance of OTUs (>0.5%) at family level from the phylum *Actinobacteria*.

The pyrosequencing of V6 hypervariable region was performed to analyze the actinobacterial community within *C. japonicas* sample from nest 1. A total of 332440 high-quality reads that distributed across 1515 OTUs were generated. Taxonomic assignment of OTUs revealed the presence of 16 known phyla, wherein the *Proteobacteria* was the most abundant phylum, followed by *Actinobacteria* as the second largest phylum (Figure 3A and Supplementary Table S2). At the family level, 40 known actinobacterial families were observed, with *Nocardioidaceae*, *Nocardiaceae*, *Dermacoccaceae*, *Intrasporangiaceae*, and *Streptomycetaceae* representing the most abundant taxa (Figure 3B and Supplementary Table S3). Cultivation-independent analyses showed more diverse actinobacterial communities than did the cultivation-dependent method. Of the 40 families detected by cultivation-independent sequencing, six families were also obtained by cultivation.

### Antifungal Activity Evaluation

**FIGURE 4:**
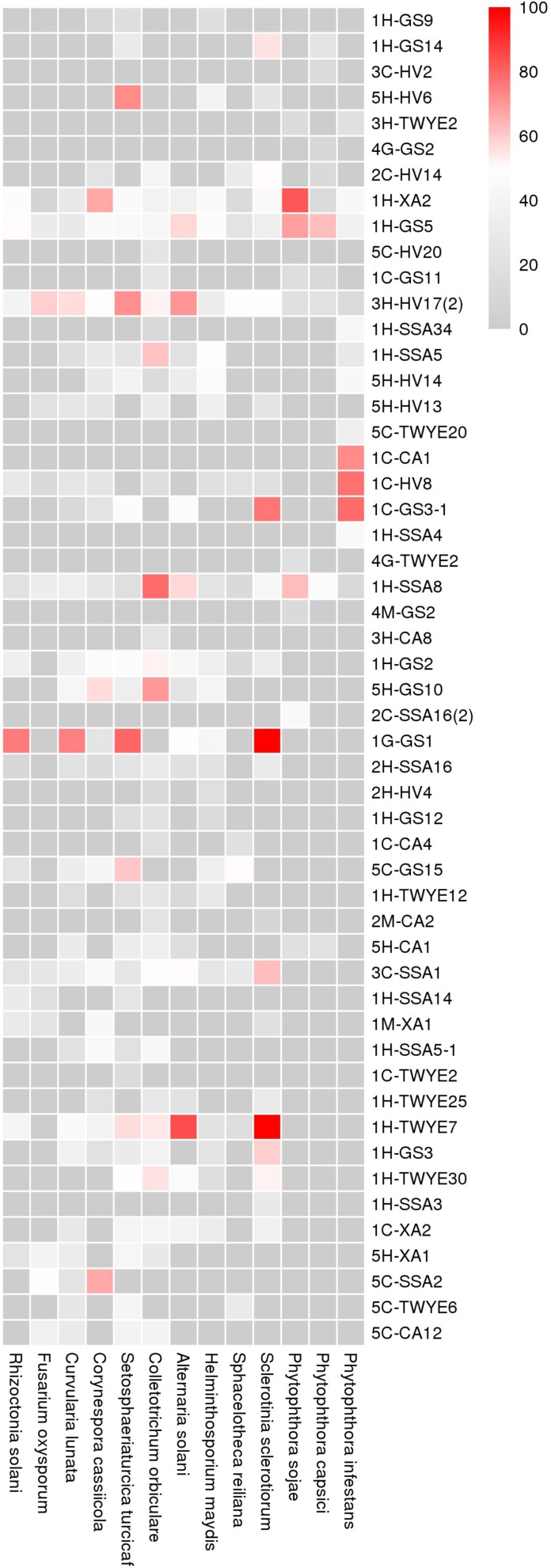
Bioassay evaluation of the antifungal activity of ant-derived actinobacteria. Boxes represent the inhibition rates (0–100%) and different colors indicate the degree of inhibition.

Fifty-six of the 107 strains (52.3%) exhibited antifungal activity against at least one of the phytopathogenic fungi tested (Figure 4). All the active strains belong to the genus *Streptomyces*. Eleven strains were found to inhibit half of the pathogens. Four strains, 1H-XA2, 1H-GS5, 1H-SSA8, and 3H-HV17(2), showed the inhibitory capability against all tested fungi. The antifungal activity against *Colletotrichum orbiculare* was the most prevalent (32 isolates, 29.9%).

### Identification of Secondary Metabolites

**FIGURE 5:**
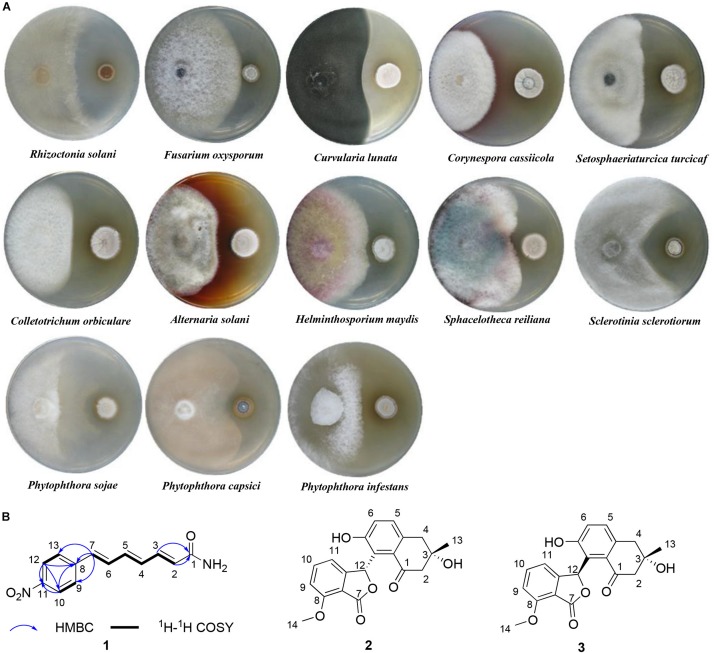
The antifungal activity and secondary metabolites of strain 1H-XA2. **(A)** The antifungal activity of strain 1H-XA2 against phytopathogenic fungi; **(B)** The structures of compounds **1–3**.

In view of the broad-spectrum antifungal activity of strain 1H-XA2 (Figure 5A), we investigated its secondary metabolites, and obtained a novel polyene amide compound (**1**), together with two known compounds, furamycins I and II (**2** and **3**). The structures were determined on the basis of spectroscopic data in this paper.

Compound **1** was obtained as yellow amorphous powder, its molecular formula C_13_H_12_N_2_O_3_ was determined by high resolution electrospray ionization mass spectrometry (HRESIMS) data (m/z 267.0740, [M+Na]^+^, calcd for 267.0740), corresponding to nine degrees of unsaturation. The ^1^H NMR and ^1^H-^1^HCOSY data (Table 1) indicated an 1,4-disubstituted phenyl group with signals at δ_H_ 7.76 (d, *J* = 8.7 Hz, 2H), 8.20 (d, *J* = 8.7 Hz, 2H). In addition, the ^1^H NMR data of **1** (Table 1) also revealed the presence of three *trans* double bonds at δ_H_ 7.30 (dd, *J* = 15.6, 10.8 Hz, H-6), 7.14 (dd, *J* = 15.0, 11.3 Hz, H-3), 6.90 (d, *J* = 15.6 Hz, H-7), 6.66 (dd, *J* = 14.7, 11.3 Hz, H-4), 6.81 (dd, *J* = 14.7, 10.8 Hz, H-5), 6.12 (d, *J* = 15.1 Hz, H-2), along with two active protons signals [δ_H_ 7.51 (brs) and 7.05 (brs)]. The ^13^C and HSQC spectra of **1** suggested thirteen carbons, which were classified into one carbonyl carbons, ten sp^2^ methine carbons and two sp^2^ quaternary carbons.

**TABLE 1:** ^1^H NMR, ^13^C NMR, COZY, and HMBC data of compound **1** in DMSO-*d*_6_.

**No.**	**δ_C_**	**δ_H_ (*J* in Hz)**	**COZY**	**HMBC**
1	166.6			
2	126.8	6.12, d (15.1)	H-3	C-4, C-1
3	138.9	7.14, dd (15.0, 11.3)	H-4, H-2	C-1, C-5
4	133.8	6.66, dd (14.7, 11.3)	H-3, H-5	C-2, C-6, C-5
5	138.0	6.81, dd (14.7, 10.8)	H-6, H-4	C-6, C-3
6	133.3	7.30, dd (15.6, 10.8)	H-7, H-5	C-4, C-8
7	132.4	6.90, d (15.6)	H-6	C-9, C-13, C-5, C-8
8	143.5			
9	127.4	7.76, d (8.7)	H-10	C-13, C-7, C-11
10	124.1	8.20, d (8.7)	H-9	C-12, C-8, C-11
11	146.3			
12	124.1	8.20, d (8.7)	H-13	C-10, C-8, C-11
13	127.4	7.76, d (8.7)	H-12	C-9, C-7, C-11
NH		7.51, s		
		7.05, s		

The ^1^H-^1^H COZY and HSQC spectrum (Supplementary Figure S1) of **1** showed one spin-coupling systems, H-2/H-3/H-4/H-5/H-6/H-7 (Figure 5B). Cross-peaks from H-7 to C-8/C-9/C-13, H-9/H-13 to C-7 were observed in the HMBC spectrum (Supplementary Figure S1), which suggested 1, 4-disubstituted phenyl group connected with olefin side chain at C-8. The HMBC cross-peaks from H-2/H-3 to C-1 (δ_C_ 166.64) revealed the presence of an amide residue, respectively. The remaining nitro functional group was determined to be substituted at C-11 by comparison of the chemical shifts with polypropionate aureothin (Ueda et al., 2007) and based on high resolution electrospray ionization mass spectrometry. The configurations of double bonds as 2*E*, 4*E*, and 6*E* were confirmed by the vicinal proton typical coupling constants (∼15 Hz). Consequently, compound **1** was elucidated as shown in Figure 5B.

Compound **2** was isolated as colorless powder, its ESI-MS spectrum showed a molecular ion peak at m/z 377 [M+Na]^+^ and 731 [2M+Na]^+^ (Supplementary Figure S2). ^1^H (400 MHz) and ^13^C (100 MHz) NMR data were shown in Table 2. Compound **2** was proved to be furammycins I by direct comparison of these data with those from the literature (Zhang S. et al., 2018).

**TABLE 2:** ^1^H NMR and ^13^C NMR data of compounds **2** and **3** in DMSO-*d*_6_.

	**2**		**3**	
**No**	**δ_C_**	**δ_H_ (*J* in Hz)**	**δ_C_**	**δ_H_ (*J* in Hz)**
1	201.0		200.5	
2	53.9	2.99, d (14.4)	53.9	2.74, s
		2.62, d (14.4)		
3	71.4		69.8	
4	43.1	2.91, d (16.3)	43.4	2.92, d (16.0)
		3.08, d (16.3)		3.02, d (16.0)
4a	133.4		133.4	
5	131.0	7.13, d (8.4)	131.2	7.15, d (8.4)
6	121.2	6.85, overlapped	121.3	6.85, d (8.4)
6a	155.6		155.8	
7	168.7		168.7	
7a	114.0		113.8	
8	157.4		157.4	
9	110.7	7.05, d (8.3)	110.6	7.05, d (8.3)
10	135.8	7.57, t (7.9)	135.8	7.55, overlapped
11	114.2	6.84, overlapped	114.2	6.80, d (7.5)
11a	152.6		152.8	
12	75.7	7.39, s	75.5	7.55, overlapped
12a	121.3		121.4	
12b	132.7		132.2	
13	29.6	1.31, s	28.8	1.27, s
14	55.7	3.90, s	55.7	3.90, s
OH-3		4.86, brs		4.88, brs
OH-7		9.61, brs		9.66, brs

Compound **3** was isolated as colorless powder. Its ESI-MS spectrum showed a molecular ion peak at m/z 377 [M+Na]^+^ and 731 [2M+Na]^+^ (Supplementary Figure S2). ^1^H (400 MHz) and ^13^C (100 MHz) NMR data were shown in Table 2. Compound **3** was proved to be furammycins II by direct comparison of these data with those from the literature (Zhang S. et al., 2018).

## Discussion

In this study, the diversity of culturable ant–associated actinobacteria obtained from three ant species is investigated. Our results have shown that the actinobacterial community is diverse and that the most abundant genus recovered is *Streptomyces*, which is consistent with previous reports (Matarrita-Carranza et al., 2017). It is worth noting that such large amounts of rare actinobacterial genera and novel taxa isolated from ant samples have not been previously reported. Instead, a single genus of actinobacteria (*Streptomyces* or *Pseudonocardia*) has often been reported to be associated with different ant species (Seipke et al., 2011; Caldera and Currie, 2012). It can be considered that the different approach employed in this study led to the successful isolation of various actinobacteria. Firstly, six different type of isolation media were used in this study. Besides CA medium that was widely used to isolate insect–associated actinobacteria (Visser et al., 2012; Matarrita-Carranza et al., 2017), several media (TWYE, SSA, XA, and HV) with relative simple nutrients that were proved to be effective for actinobacteria isolation were used in this study (Qin et al., 2012). Especially, HV medium significantly increased the recovery of isolates as well as rare actinobacterial genera, which resemble with soil-derived or endophytic actinobacteria isolation (Matsukawa et al., 2007; Kaewkla and Franco, 2013). Moreover, actinobacteria from different body segments of the ants (cuticle, head, thorax, and gaster) were investigated. Previous studies have focused on actinobacteria from the cuticle (Currie et al., 1999; Zucchi et al., 2011; Caldera and Currie, 2012). Although large amounts of isolates were also obtained in this segment, interestingly, the head segment yielded the highest number of isolates in the present study. This finding could potentially be due to the infrabuccal pocket, a specialized pouch in the oral cavity of the ants, which is known to be used for cleaning themselves, their nestmates and brood (Hölldobler and Wilson, 1990). Early results indicated that the pocket of *C. japonicas* harbored a high abundance of actinobacteria strains (Zhang et al., 2016; Zhang K.X. et al., 2018).

Out of the five nests sampled, the ant sample from nest 4 yielded the fewest number of isolates. The ant species from nests 1, 2, and 4 were the same. The only difference is that nests **1** and **2** located in the soil environment, whereas nest 4 was built on a tree. This result could be owing to the fact that solid food and other materials with environmental microorganisms will be accumulated in the infrabuccal pocket, which led to the potential for some of the isolates we obtained being environmental contaminants. Therefore, fewer isolates from nest 4 observed here could be explained by less abundant microorganisms existing in plant tissue compared with soil environment.

Considering the limitations of isolation methods, the culture-independent approach was employed to accurately evaluate the composition of ant-derived actinobacterial community. A total of 40 known actinobacterial families were detected in the ant sample, whereas only six families were isolated by culturing. This result provides the impetus for the development of new methods or strategies to obtain more diverse antinobacteria from ants in the future studies. Taxonomic classification of the OTU-representative sequences to family level showed that *Nocardioidaceae* (15.7%) and *Nocardiaceae* (12.5%) largely dominate the ant host actinobacterial community, whereas *Streptomycetaceae* observed as the most dominant cultivated family was found in lower abundance (6.6%). Similar results were also reported in the soil environment, where *Streptomyces* belonging to the family *Streptomycetaceae* was the predominant genus of culturable actinobacteria, but it was not the largest community detected by culture-independent technique (Lundberg et al., 2012). It might be because the isolation media used in this study presents a bias that favored the growth of *Streptomyces* strains. Other studies also demonstrated that *Streptomyces* was the most commonly isolated genus from soil, plant and insect environments using these media, although many rare actinobacteria genera were also recovered (Matsukawa et al., 2007; Qin et al., 2012; Kaewkla and Franco, 2013; Matarrita-Carranza et al., 2017). The interaction between members of the genus *Pseudonocardia* in the family *Pseudonocardiaceae* and fungus-growing ants has been extensively reported (Currie et al., 1999; Andersen et al., 2013). Members of the family *Pseudonocardiaceae* were not obtained by cultivation. However, the relatively high abundance of the family (3.1%) was detected by high-throughput sequencing method.

To validate that ant-derived actinobacteria represent a source of antimicrobials with activity against phytopathogenic fungi, 107 different strains were conducted *in vitro* antagonism assays using 13 different phytopathogenic fungi. Our results indicated that a high percentage of strains (52.3%) possessed the capacity to inhibit fungi. There is also evidence that insect-associated actinobacteria showed significantly greater antifungal activity compared to soil and plant-associated actinobacteria (Chevrette et al., 2019). All the active strains belong to the genus *Streptomyces* through 16S rRNA gene sequence analysis. Indeed, it is not surprising that members of this taxon encompassed the most active strains, as revealed by previous studies (Verma et al., 2009; Liu et al., 2019). Notably, high proportions of active strains (52%, 29 strains) were obtained from the head sample. This result could potentially be correlated with the infrabuccal pocket, where fungus-growing ants have a symbiotic association with actinobacteria that inhibit the parasitic fungus (*Escovopsis* sp.) (Little et al., 2006).

Actinobacteria from insect microbiomes are a rich source of antifungal natural products and a number of compounds with unique structures were identified (Van Arnam et al., 2018; Chevrette et al., 2019). Our chemical investigation of four ant-derived *Streptomyces* strains led to the discovery of eight known compounds and two new polyketide compounds (Liu et al., 2016, 2017; Cao et al., 2017; Ye et al., 2017; Jiang et al., 2018). Among the ten compounds, amphotericin, and kanchanamycin are known as excellent antifungal agents (Fiedler et al., 1996; Hamill, 2013; Zhang et al., 2019). In this report, we investigated the secondary metabolites from ant-derived *Streptomyces* strain 1H-XA2 with broad-spectrum antifungal activity, which resulted in the discovery of furamycins I and II (2 and 3), as well as a novel polyene amide compound (**1**). Furamycins I and II have been reported to be produced by a marine *Streptomyces* species and to show strong antifungal activity against *Candida albicans*, *Colletortrichum gloeosporiodes*, and *Penicillium marneffei* (Zhang S. et al., 2018). Polyene compounds with antifungal activity were also recovered from insect host *Streptomyces* (Oh et al., 2009b, 2011). However, compound **1** did not show any antifungal or antibacterial activity (data not shown). Previous study showed that its structural analogs, trichostatin A and BL1521, could inhibit proliferation and induce apoptosis in neuroblastoma cells (de Ruijter et al., 2004). Therefore, further bioactive assays to define its functional role is under way.

## Conclusion

Ant-derived actinobacterial community structure was analyzed using both culture-dependent and -independent methods. Ants harbored abundant and diverse communities of actinobacteria, including many rare actinobacteria and novel taxa. Antifungal activity assays showed that high percentage of the isolates had antagonistic activity agaist phytopathogenic fungi. In addition, two known compounds and one new compound were identified. These results suggest that ant-derived actinobacteria represent a promising and underexplored resource for exploring novel agricultural antifungals.

## Data Availability Statement

Publicly available datasets were analyzed in this study. This data can be found here: The obtained 16S rRNA gene sequences were deposited in the GenBank database under accession numbers KP784763–KP784808, KX777578–KX777633, KX977397–KX977399, and KR261651–KR261652, respectively. High-throughput sequencing data can be retrieved from the NCBI Short Read Archive under accession number SRR8061509.

## Author Contributions

CL and JZ designed the experiments. CL, ZW, ZY, JZ, XZ, PC, and XG performed the experiments. CL analyzed the data and prepared the manuscript. WX reviewed the manuscript.

## Conflict of Interest

The authors declare that the research was conducted in the absence of any commercial or financial relationships that could be construed as a potential conflict of interest.
