# Supplementary material for: Community Composition, Antifungal Activity and Chemical Analyses of Ant-Derived Actinobacteria
Source: Front Microbiol. 2020 Feb 11;11:201. doi: 10.3389/fmicb.2020.00201 (PMC7026373; doi:10.3389/fmicb.2020.00201)
Supplement: Supplementary file 1 [file Data_Sheet_1.pdf]

Supporting information

Community Composition, Antifungal Activity and Chemical Analyses of Ant-derived Actinobacteria

Zhiyan Wang[1,2]†, Zhiyin Yu[1,2]†, Junwei Zhao[1]†, Xiaoxin Zhuang[1], Peng Cao[1], Chongxi Liu[1,2*], Wensheng Xiang[1,3*]

[1]Key Laboratory of Agricultural Microbiology of Heilongjiang Province, Northeast Agricultural University, Harbin 150030, China

[2]State Key Laboratory of Phytochemistry and Plant Resources in West China, Kunming Institute of Botany, Chinese Academy of Sciences, Kunming 650201, China

[3]State Key Laboratory for Biology of Plant Diseases and Insect Pests, Institute of Plant Protection, Chinese Academy of Agricultural Sciences, Beijing 100193, China

*Correspondence: Chongxi Liu and Wensheng Xiang

E-mail: xizi-ok@163.com, xiangwensheng@neau.edu.cn

†These authors have contributed equally to this work

Running title:“Actinobacteria from Ants”

Table S1 Taxonomic distribution of ant-derived actinobacterial isolates from different nests, body parts and isolation media.

Isolate	Closest Type Strain and Similarity	Nest	Body Part	Medium
1H-GS3	*Streptomyces ossamyceticus* (99.9%)	1, 5	Head, Cuticle	GS, TWYE
1H-SSA4	*Streptomyces ossamyceticus* (98.6%)	1	Head	SSA
1H-TWYE36	*Streptomyces stelliscabiei* (98.6%)	1	Head	TWYE
1H-CA9	*Streptomyces scabiei* (99.7%)	1, 4	Head, Thorax	CA, XA
1C-GS8	*Streptomyces phaeochromogenes* (99.2%)	1, 3	Cuticle, Head, Thorax	GS, HV, CA, SSA
1H-SSA6	*Streptomyces rectiviolaceus* (99.9%)	1	Head	SSA
1H-SSA8	*Streptomyces ramulosus* (99.2%)	1	Head	SSA
1H-TWYE2	*Streptomyces durmitorensis* (99.7%)	1, 3	Head	TWYE, GS
1C-CA1	*Streptomyces resistomycificus* (100%)	1	Cuticle	CA
1C-TWYE2	*Streptomyces xanthophaeus* (99.9%)	1	Cuticle, Head	TWYE, CA
1H-TWYE12	*Streptomyces colombiensis* (99.8%)	1	Head	TWYE
1H-GS2	*Streptomyces candidus* (99.4%)	1	Head	GS
1H-SSA34	*Streptomyces spiroverticillatus* (99.3%)	1, 2, 5	Head, Cuticle	SSA, HV, XA, TWYE, CA, GS
1C-CA4	*Streptomyces fulvissimus* (99.9%)	1	Cuticle	CA
1C-GS11	*Streptomyces puniceus* (99.9%)	1	Cuticle	GS
1H-GS12	*Streptomyces globisporus* (99.9%)	1	Head	GS
1H-TWYE23	*Streptomyces gardneri* (99.6%)	1, 3, 4	Head, Cuticle, Gaster	TWYE, GS, CA
1H-GS9	*Streptomyces scopuliridis* (98.8%)	1	Head	GS
1H-XA2	*Streptomyces netropsis* (99.7%)	1	Head	XA
1C-XA2	*Streptomyces netropsis* (99.7%)	1	Cuticle	XA

1C-GS3-1	*Streptomyces sioyaensis* (99.7%)	1, 5	Cuticle, Head	GS, SSA, HV, TWYE
1C-HV8	*Streptomyces sioyaensis* (99.9%)	1, 2, 4	Cuticle, Thorax, Gaster	HV, CA, GS
1H-GS14	*Streptomyces sioyaensis* (99.6%)	1	Head	GS
1G-GS1	*Streptomyces nigrescens* (99.8%)	1, 2	Gaster, Head	GS, HV
1H-TWYE7	*Streptomyces nigrescens* (99.9%)	1	Head	TWYE
1H-SSA3	*Streptomyces angustmyceticus* (99.7%)	1	Head, Gaster	SSA, TWYE
1H-TWYE25	*Streptomyces angustmyceticus* (99.7%)	1	Head	TWYE
1H-SSA5	*Streptomyces angustmyceticus* (99.9%)	1	Head	SSA, GS
1H-SSA14	*Streptomyces fradiae* (100%)	1	Head	SSA
1H-GS5	*Streptomyces spectabilis* (99.9%)	1	Head	GS
1H-TWYE14	*Streptomyces rubrogriseus* (99.4%)	1	Head	TWYE
1H-TWYE10	*Streptomyces rochei* (99.9%)	1	Head	TWYE
1H-TWYE30	*Streptomyces luteogriseus* (99.9%)	1	Head	TWYE
1M-XA1	*Streptomyces albidoflavus* (99.7%)	1, 2, 3, 4	Thorax, Head, Cuticle	XA, CA, HV, TWYE
1H-SSA5(2)	*Streptomyces hyalinus* (99.7%)	1	Head	SSA
1C-HV13	*Streptosporangium nanhuense* (99.7%)	1	Cuticle	HV
1C-HV12	*Promicromonospora kermanensis* (99.5%)	1	Cuticle	HV
1H-HV4	*Nocardia salmonicida* (97.4%)	1	Head	HV
1C-GS7	*Verrucosispora rhizosphaerae* (99.4%)	1	Cuticle	GS
1C-CA6	*Phytohabitans houttuyneae* (99.8%)	1	Cuticle	CA
1G-HV2	*Micromonospora taraxaci* (99.7%)	1	Gaster	HV
2C-HV14	*Streptomyces deccanensis* (98.9%)	1, 2	Cuticle, Head, Gaster	HV, SSA, CA, GS
2M-TWYE1	*Streptomyces ederensis*	2	Thorax	TWYE

	(99.2%)			
2C-SSA16-1	*Streptomyces umbrinus* (99.0%)	2, 4, 5	Cuticle, Head	SSA, GS, CA, HV
2C-SSA7	*Streptomyces tauricus* (99.5%)	2, 5	Cuticle	SSA, GS, TWYE
2H-SSA3	*Streptomyces pseudovenezuelae* (99.1%)	2	Head	SSA
2M-CA2	*Streptomyces fulvissimus* (99.9%)	2	Thorax	CA
2H-HV12	*Streptomyces tauricus* (100%)	1, 2, 4, 5	Head, Cuticle, Thorax	HV, CA, XA, SSA, GS, TWYE
2H-HV4	*Streptomyces badius* (100%)	2, 5	Head, Cuticle	HV, SSA
2H-TWYE14	*Streptomyces niveus* (99.4%)	2, 4	Head, Cuticle, Thorax, Gaster	TWYE, GS, SSA
2H-SSA16	*Streptomyces albidoflavus* (99.9%)	1, 2	Head, Cuticle	SSA
2C-HV3	*Microbispora bryophytorum* (99.9%)	2	Cuticle	HV
2M-SSA4	*Nocardia salmonicida* (99.1%)	2	Thorax	SSA
2C-HV13	*Micromonospora taraxaci* (99.9%)	2, 4	Cuticle, Head	HV, TWYE
2M-SSA2	*Micromonospora inaquosa* (99.9%)	2, 3	Thorax, Cuticle	SSA, TWYE
2C-HV13-1	*Micromonospora taraxaci* (99.9%)	2	Cuticle	HV
2C-SSA2	*Micromonospora taraxaci* (99.9%)	2	Cuticle	SSA
2C-CA12	*Micromonospora taraxaci* (99.9%)	2	Cuticle	CA
2G-CA3	*Micromonospora taraxaci* (99.7%)	2	Gaster	CA
2C-HV12	*Micromonospora taraxaci* (99.4%)	2	Cuticle	HV
3H-XA8	*Streptomyces fulvissimus* (99.6%)	3	Head, Thorax	XA, SSA, TWYE
3H-GS5	*Streptomyces fulvissimus* (100%)	3	Head	GS, HV
3H-HV17(1)	*Streptomyces setonii* (99.9%)	3, 4, 5	Head, Cuticle	HV, XA, GS
3H-HV6	*Streptomyces puniceus* (99.9%)	2, 3	Head, Gaster	HV, CA
3H-HV1	*Streptomyces puniceus* (99.9%)	3	Head	HV, GS

3C-HV2	*Streptomyces puniceus* (99.2%)	3	Cuticle, Head, Thorax	HV, GS, XA
3H-TWYE2	*Streptomyces tanashiensis* (99.4%)	3, 4	Head, Thorax, Gaster	TWYE, GS, HV
3C-SSA1	*Streptomyces sporoclivatus* (99.9%)	3, 5	Cuticle, Head	SSA, GS
3H-HV17(2)	*Streptomyces gougerotii* (98.8%)	3	Head	HV
3H-CA8	*Streptomyces rochei* (100%)	1, 3, 5	Head, Cuticle	CA, XA, TWYE
3H-GS17	*Actinocorallia glomerata* (98.0%)	3	Head	GS, TWYE
3C-HV12	*Nocardia salmonicida* (99.2%)	3	Cuticle	HV
3C-CA11	*Micromonospora tulbaghiae* (99.9%)	3	Cuticle, Thorax	CA, SSA
3H-HV25	*Micromonospora chalcea* (99.3%)	3, 5	Head	HV, GS
3C-HV5	*Micromonospora auratinigra* (99.2%)	3, 5	Cuticle, Gaster	HV, TWYE, CA
3C-HV4	*Micromonospora taraxaci* (99.2%)	3	Cuticle	HV
3C-GS13-1	*Micromonospora taraxaci* (99.6%)	1, 2, 3, 5	Cuticle	GS, TWYE, CA, HV
4G-GS2	*Streptomyces ossamyceticus* (100%)	4, 5	Head, Thorax, Gaster	GS, HV, SSA
4H-CA5	*Streptomyces rhizosphaerihabitans* (99.6%)	2, 4, 5	Head, Cuticle	CA, XA
4M-GS2	*Streptomyces seoulensis* (99.5%)	1, 4	Thorax, Cuticle	GS, XA
4G-TWYE2	*Streptomyces seoulensis* (99.5%)	2, 4, 5	Head, Cuticle, Gaster	TWYE, SSA, GS
4M-GS8	*Streptomyces cirratus* (99.9%)	4	Thorax	GS
4H-TWYE9	*Streptomyces neopeptinius* (99.0%)	4	Head	TWYE
4M-CA7	*Micromonospora rifamycinica* (99.7%)	1, 4	Thorax, Head	CA, GS
5C-GS1	*Streptomyces ossamyceticus* (99.9%)	3, 5	Cuticle, Head	GS, TWYE
5C-GS15	*Streptomyces ossamyceticus* (99.9%)	5	Cuticle	GS
5C-TWYE6	*Streptomyces cinereoruber* (99.6%)	5	Cuticle	TWYE, SSA
5H-XA1	*Streptomyces rectiviolaceus*	5	Head	XA

	(99.9%)			
5H-CA1	*Streptomyces tauricus* (99.9%)	2, 5	Head, Cuticle	CA, SSA, GS
5C-HV24	*Streptomyces pseudovenezuelae* (99.2%)	5	Cuticle	HV, SSA
5C-TWYE20	*Streptomyces canus* (99.7%)	5	Cuticle	TWYE, GS, XA
5H-CA17	*Streptomyces xanthophaeus* (99.9%)	1, 2, 3, 5	Head, Cuticle, Thorax	CA, GS, XA, SSA
5H-CA12	*Streptomyces xanthophaeus* (99.9%)	5	Head, Cuticle	CA, HV
5H-HV13	*Streptomyces cirratus* (99.9%)	5	Head	HV
5H-HV6	*Streptomyces netropsis* (99.9%)	5	Head	HV
5H-GS10	*Streptomyces angustmyceticus* (99.7%)	2, 5	Head	GS, SSA
5C-SSA2	*Streptomyces angustmyceticus* (99.7%)	4, 5	Cuticle	SSA, XA
5H-HV14	*Streptomyces angustmyceticus* (99.7%)	1, 5	Head	HV, CA
5H-XA2	*Streptomyces angustmyceticus* (99.7%)	5	Head	XA
5H-CA11	*Streptomyces hyalinus* (98.9%)	5	Head	CA
5C-SSA15	*Nonomuraea guangzhouensis* (99.7%)	5	Cuticle	SSA
5C-CA9	*Micromonospora inositola* (99.5%)	5	Cuticle	CA
5G-GS1	*Micromonospora rifamycinica* (99.9%)	5	Gaster	GS
5C-CA12	*Micromonospora rifamycinica* (99.9%)	5	Cuticle	CA
5C-CA4	*Micromonospora carbonacea* (99.8%)	5	Cuticle	CA
5C-GS5	*Micromonospora taraxaci* (99.9%)	5	Cuticle	GS
5C-HV20	*Streptomyces glauciniger* (99.9%)	5	Cuticle	HV

Table S2. The relative abundance of OTUs at phylum level.

**Phylum**	**Relative abundance (%)**
Proteobacteria	96.30
Actinobacteria	2.81
Firmicutes	0.26
Chloroflexi	0.17
Gemmatimonadetes	0.13
Acidobacteria	0.13
Planctomycetes	0.062
Verrucomicrobia	0.057
Cyanobacteria	0.041
Nitrospirae	0.026
Armatimonadetes	0.0059
Chlorobi	0.0051
Tenericutes	0.0034
Chlamydiae	0.0034
Fibrobacteres	0.0017
Bacteroidetes	0.00084

Table S3. The relative abundance of OTUs at family level from the phylum *Actinobacteria*

Family	Relative abundance (%)
Nocardioidaceae	15.67
Nocardiaceae	12.54
Dermacoccaceae	7.61
Intrasporangiaceae	7.08
Streptomycetaceae	6.59
Mycobacteriaceae	6.25
Micrococcaceae	5.57
Gaiellaceae	5.46
Thermomonosporaceae	4.86
Microbacteriaceae	3.95
Propionibacteriaceae	3.65
Micromonosporaceae	3.58
Pseudonocardiaceae	3.05
Geodermatophilaceae	2.79
Sporichthyaceae	1.81
Tsukamurellaceae	1.47
Cellulomonadaceae	1.28
Patulibacteraceae	1.17
Streptosporangiaceae	0.87
Nakamurellaceae	0.83
Gordoniaceae	0.64
Corynebacteriaceae	0.53
Rubrobacteraceae	0.49
Kineosporiaceae	0.45
Nocardiopsaceae	0.34
Actinosynnemataceae	0.30
Bogoriellaceae	0.23
Solirubrobacteraceae	0.23
Frankiaceae	0.15
Promicromonosporaceae	0.11
Iamiaceae	0.075
Sanguibacteraceae	0.075
Microthrixaceae	0.038
Brevibacteriaceae	0.038
Dietziaceae	0.038
Nitriliruptoraceae	0.038
Dermabacteraceae	0.038
Coriobacteriaceae	0.038
Euzebyaceae	0.038
Conexibacteraceae	0.038

FIGURE CAPTIONS

Figure S1. $^{\mathbf{1}}$H-NMR, ^{13}C-NMR, ^{1}H-^{1}H COSY, HSQC, HMBC and HRESI spectra of compound **1**. **(a)** $^{\mathbf{1}}$H-NMR (400 MHz) spectrum in DMSO-d_6; **(b)** ^{13}C-NMR (100 MHz) spectrum in DMSO-d_6; **(c)** ^{1}H-^{1}H COSY (400 MHz) spectrum in DMSO-d_6; **(d)** HSQC (400 MHz) spectrum in DMSO-d_6; **(e)** HMBC (400 MHz) spectrum in DMSO-d_6; **(f)** HRESI spectrum.

Figure S2. $^{\mathbf{1}}$H-NMR, ^{13}C-NMR and ESI spectra of compound **2** and **3**. **(a)** ^{1}H-NMR (400 MHz) spectrum of compound **2** in DMSO-d_6; **(b)** ^{13}C-NMR (400 MHz) spectrum of compound **2** in DMSO-d_6; **(c)** ESI^{+} spectrum of compound **2**; **(d)** ^{1}H-NMR (400 MHz) spectrum of compound **3** in DMSO-d_6; **(e)** ^{13}C-NMR (400 MHz) spectrum of compound **3** in DMSO--d_6; **(f)** ESI^{+} spectrum of compound **3**.

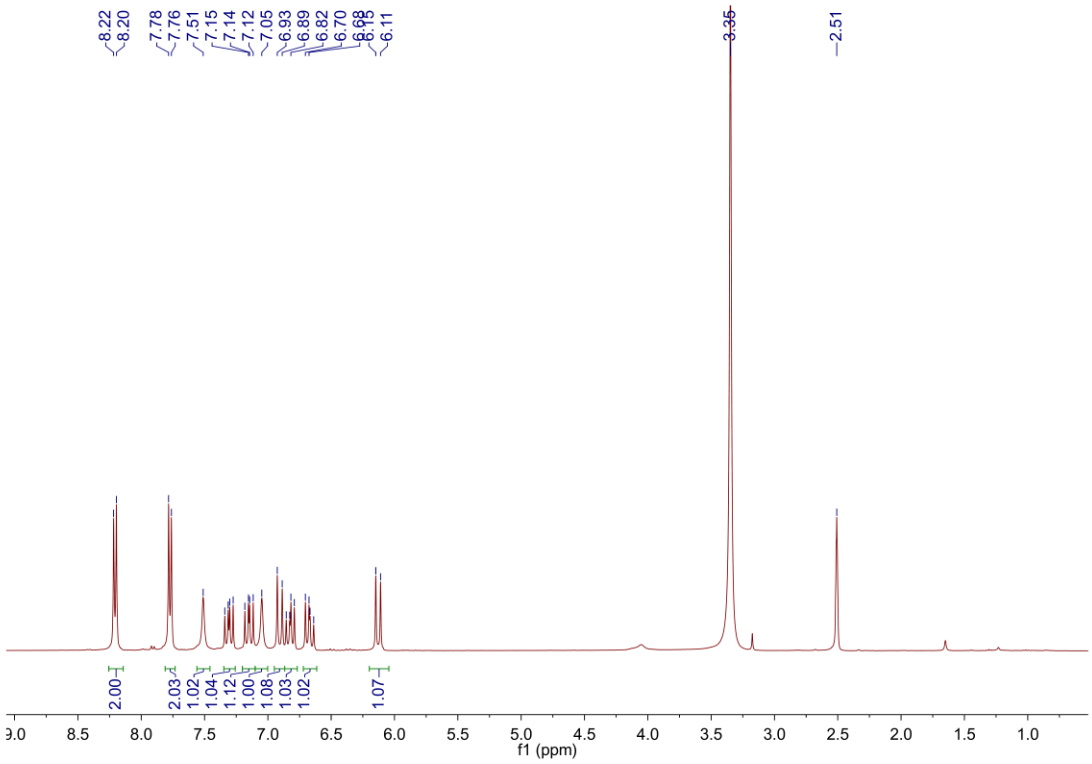

Figure S1a

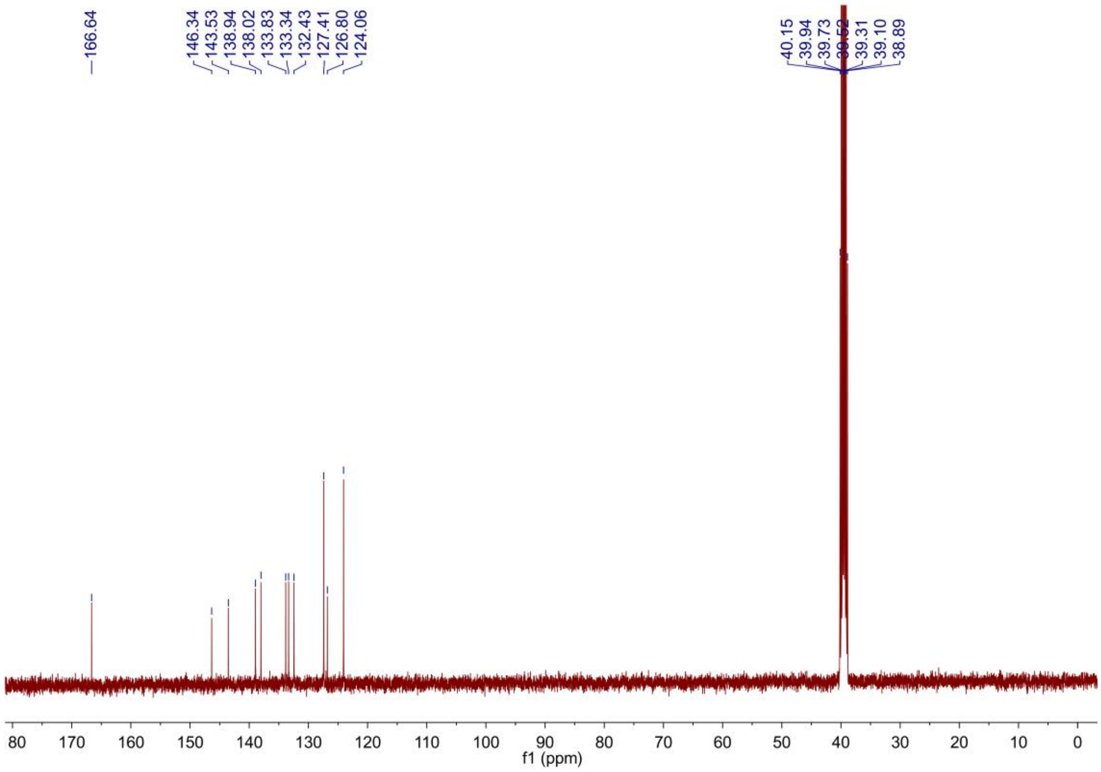

Figure S1b

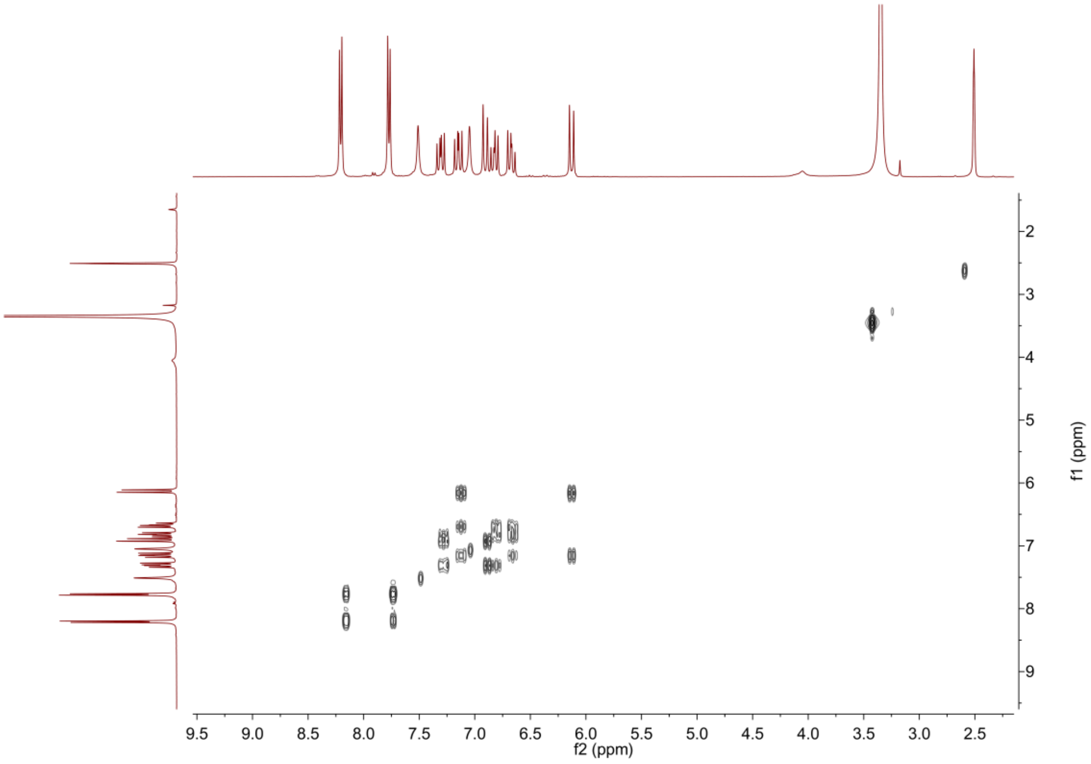

Figure S1c

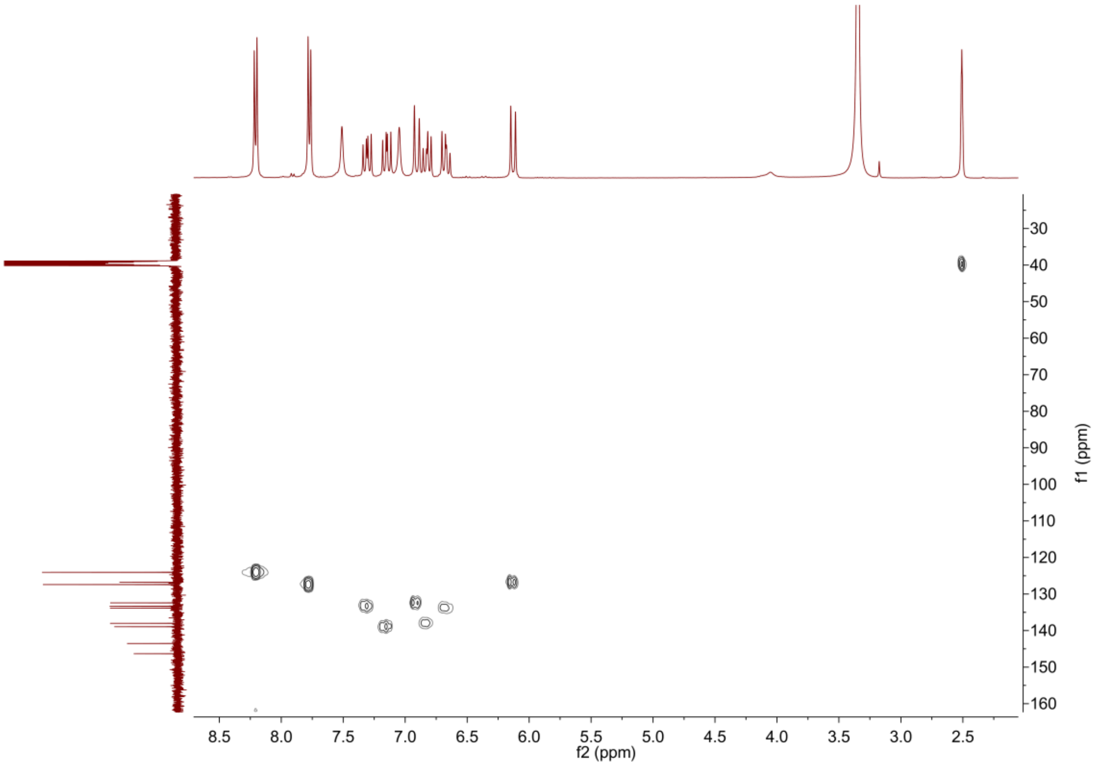

Figure S1d

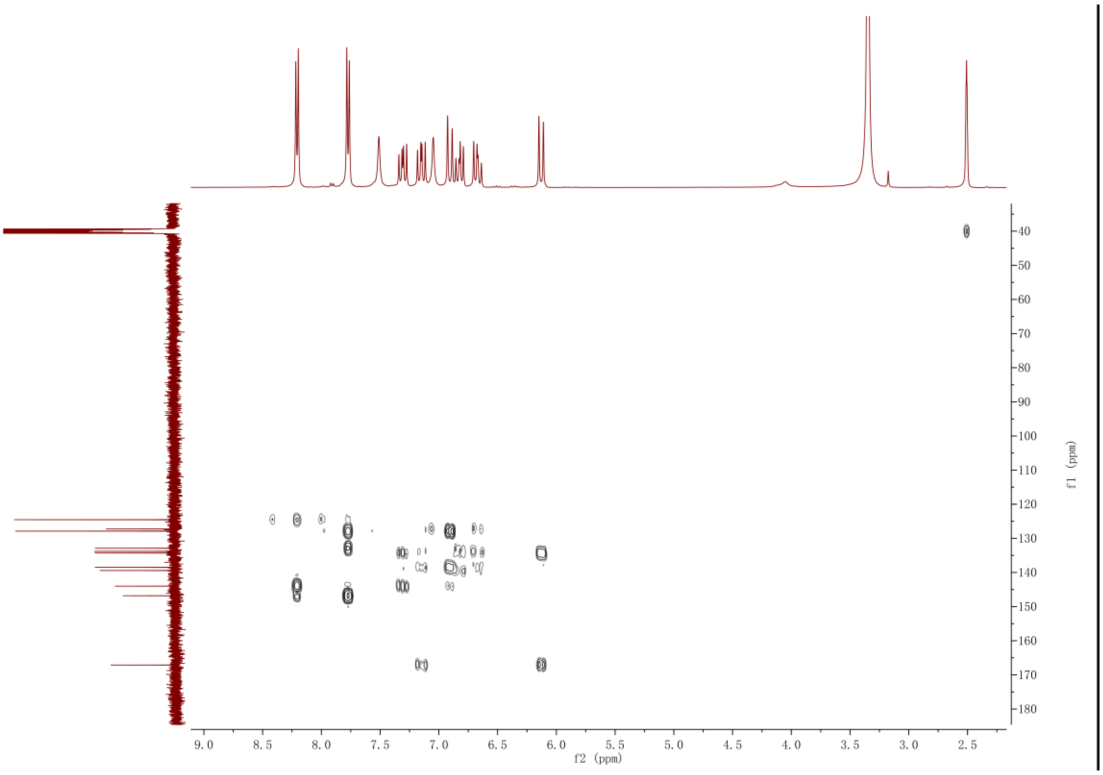
f1 (ppm)
f2 (ppm)
9.0
8.5
8.0
7.5
7.0
6.5
6.0
5.5
5.0
4.5
4.0
3.5
3.0
2.5
40
50
60
70
80
90
100
110
120
130
140
150
160
170
180

Figure S1e

Data File: E:\DATA\2018\1213\HSXC909.lcd

Elmt	Val.	Min	Max	Elmt	Val.	Min	Max	Elmt	Val.	Min	Max	Elmt	Val.	Min	Max	Use Adduct
H	1	10	100	F	1	0	0	S	2	0	0	Ag	1	0	0	Na
C	4	10	100	Na	1	0	0	Cl	1	0	0	I	3	0	0	
N	3	0	20	Mg	2	0	0	Se	2	0	0					
O	2	0	10	Si	4	0	0	Br	1	0	0					

Error Margin (ppm): 5
HC Ratio: unlimited
Max Isotopes: all
MSn Iso RI (%): 75.00

DBE Range: -2.0 - 100.0
Apply N Rule: yes
Isotope RI (%): 1.00
MSn Logic Mode: OR

Electron Ions: both
Use MSn Info: yes
Isotope Res: 10000
Max Results: 10

Event#: 1 MS(E+) Ret. Time : 0.373 -> 0.387 Scan# : 57 -> 59

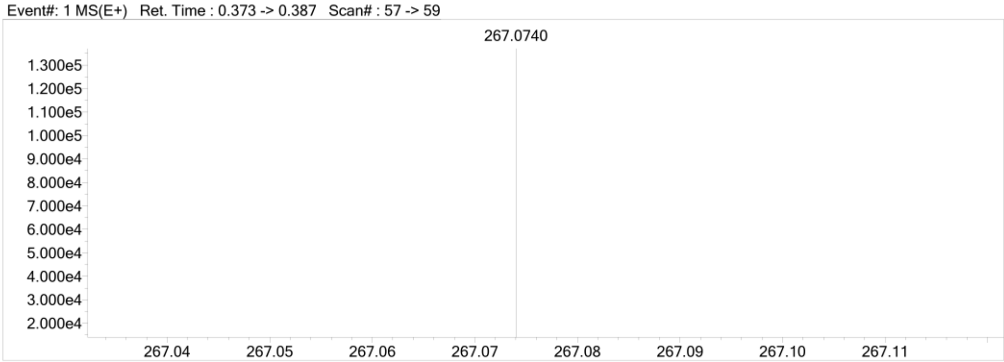

Measured region for 267.0740 m/z

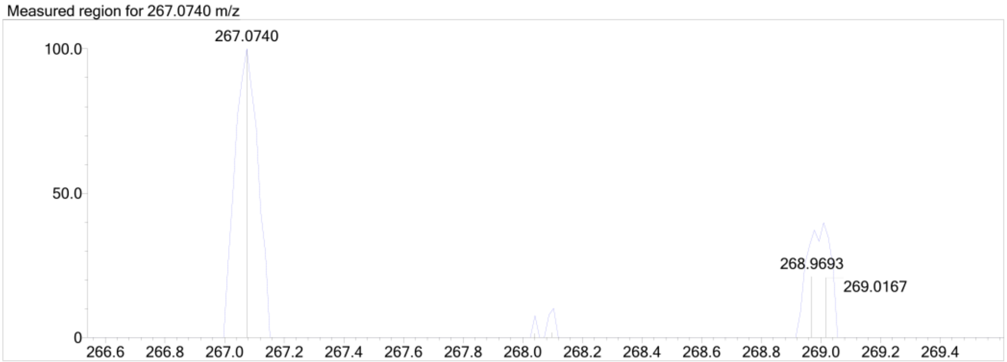

C13 H12 N2 O3 [M+Na]+ : Predicted region for 267.0740 m/z

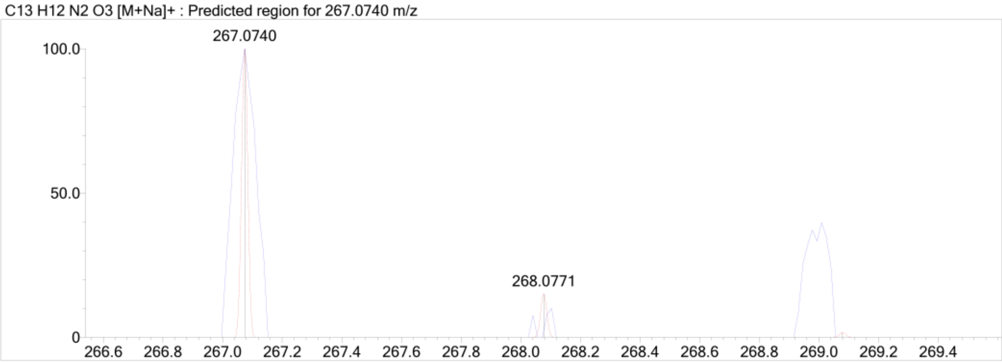

Formula (M)	Ion	Meas. m/z	Pred. m/z	Df. (mDa)	Df. (ppm)	DBE
C13 H12 N2 O3	[M+Na]+	267.0740	267.0740	-0.0	0.00	9.0

Figure S1f

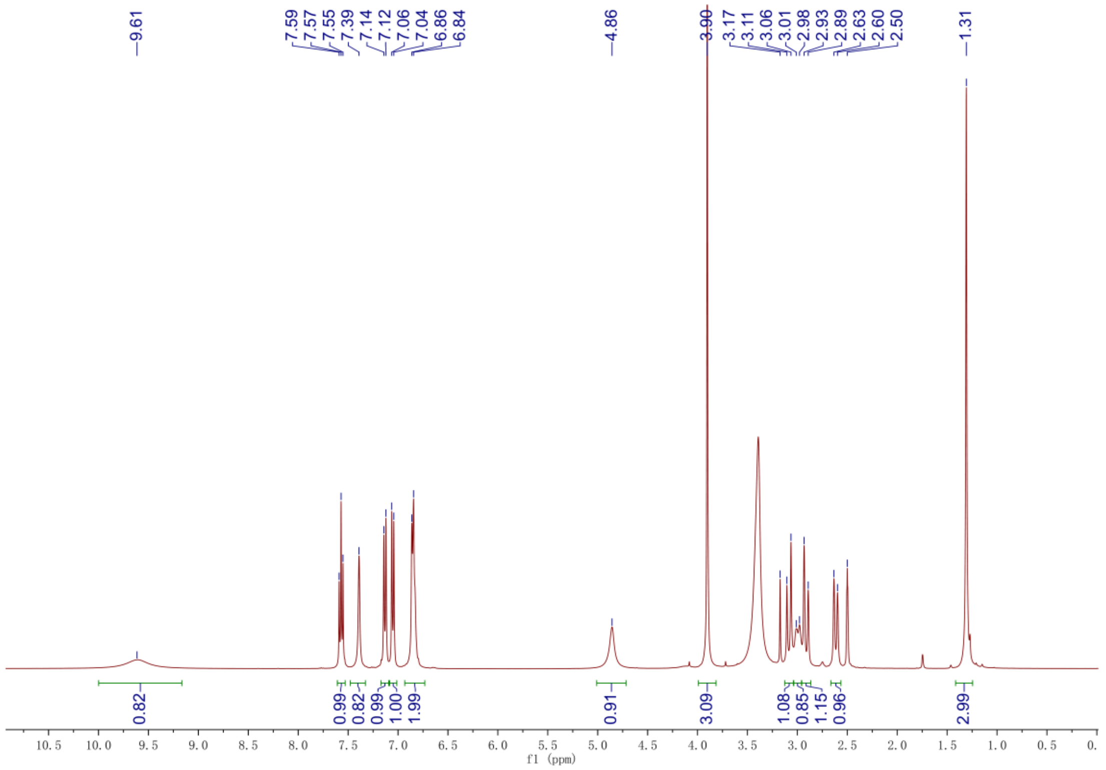

Figure S2a

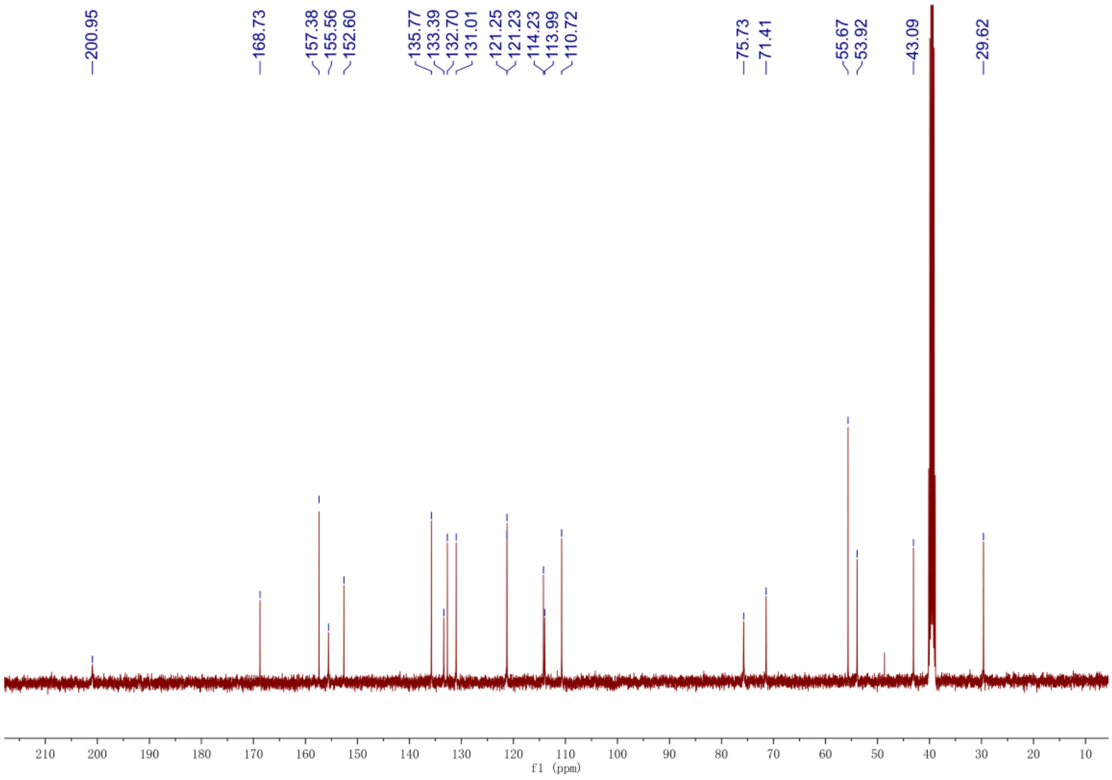

Figure S2b

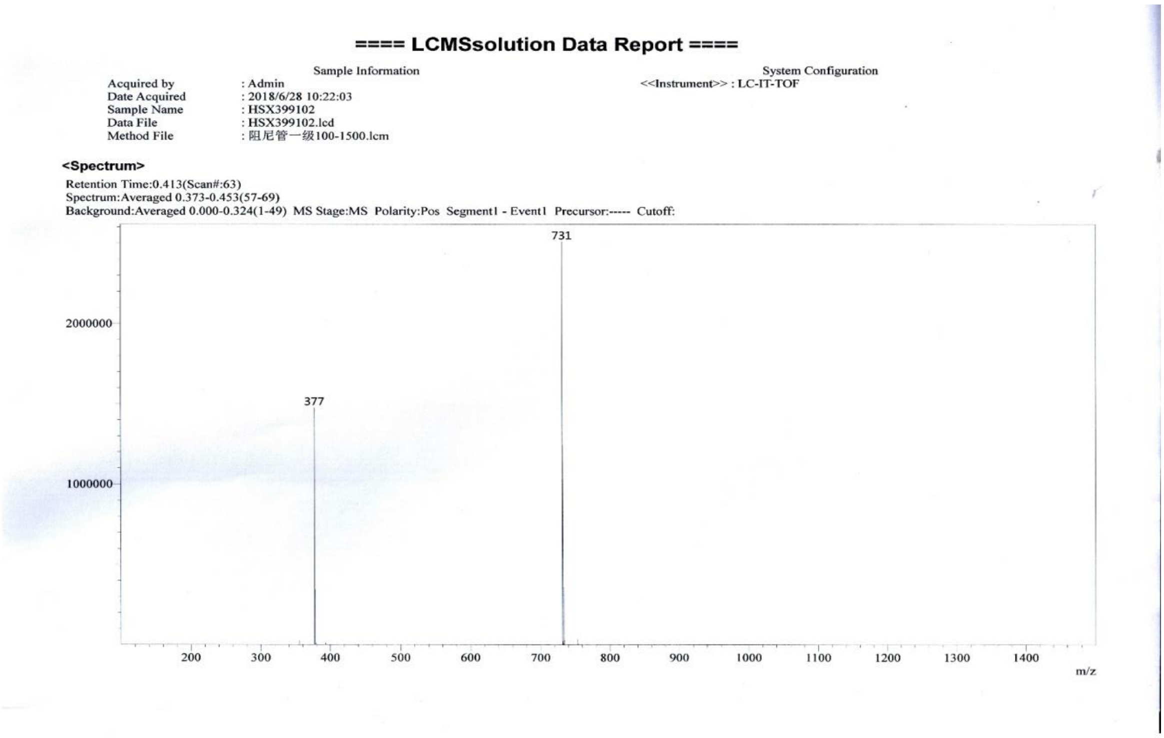
==== LCMSsolution Data Report ====
Sample Information
Acquired by : Admin
Date Acquired : 2018/6/28 10:22:03
Sample Name : HSX399102
Data File : HSX399102.lcd
Method File : 阻尼管一级100-1500.lcm
System Configuration
<<Instrument>> : LC-IT-TOF
<Spectrum>
Retention Time:0.413(Scan#:63)
Spectrum:Averaged 0.373-0.453(57-69)
Background:Averaged 0.000-0.324(1-49) MS Stage:MS Polarity:Pos Segment1 - Event1 Precursor:----- Cutoff:
731
377
2000000
1000000
200
300
400
500
600
700
800
900
1000
1100
1200
1300
1400
m/z

Figure S2c

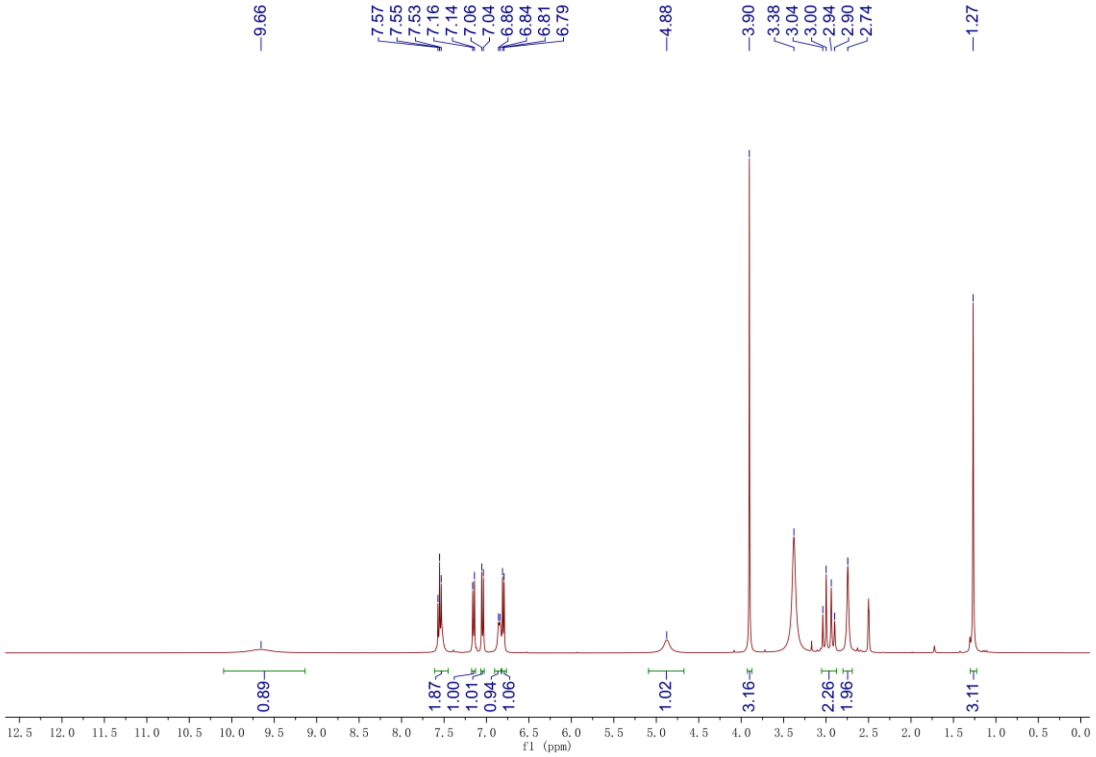

Figure S2d

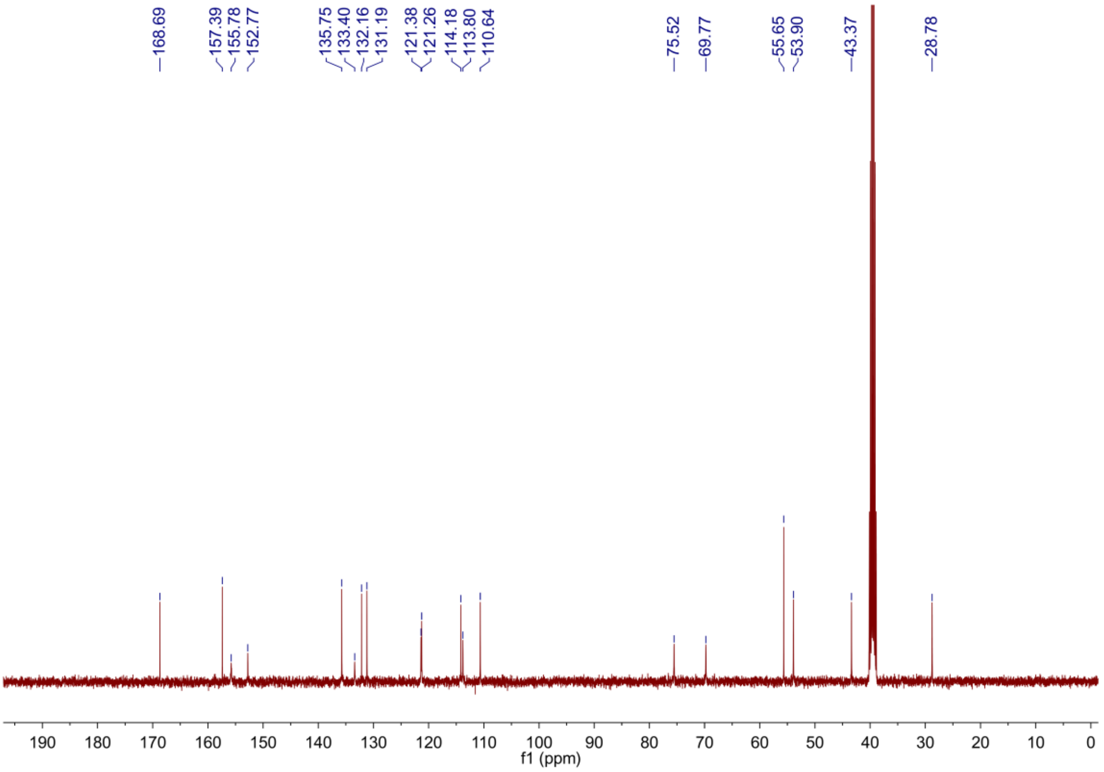

Figure S2e

==== LCMSsolution Data Report ====

Sample Information

Acquired by	: Admin
Date Acquired	: 2018/6/28 10:23:39
Sample Name	: HSX399103
Data File	: HSX399103.lcd
Method File	: 阻尼管一级100-1500.lcm

System Configuration

<<Instrument>> : LC-IT-TOF

<Spectrum>

Retention Time:0.493(Scan#:76)
Spectrum:Averaged 0.293-0.707(45-107)
Background:Averaged 0.000-0.330(1-51) MS Stage:MS Polarity:Pos Segment1 - Event1 Precursor:----- Cutoff:

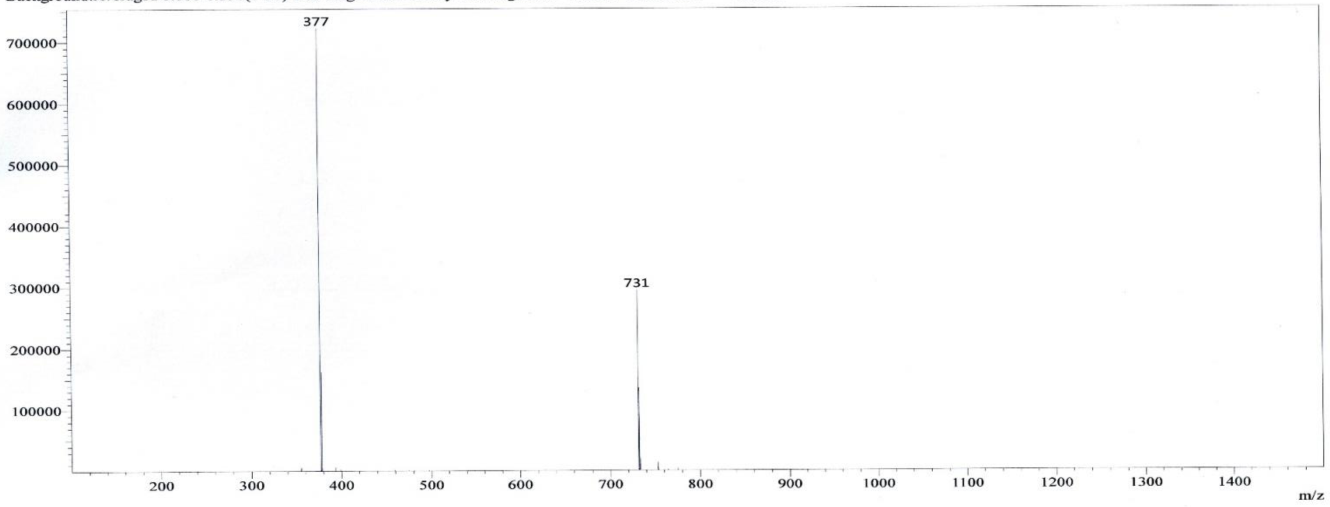

Figure S2f
